# Staphylococcal enterotoxin B administration in pregnant rats alters the splenic lymphocyte response in adult offspring rats

**DOI:** 10.1186/s12866-016-0921-2

**Published:** 2017-01-03

**Authors:** Ping Zhou, Xin-sheng Zhang, Zhi-ben Xu, Shu-xian Gao, Qing-wei Zheng, Ming-zhu Xu, Lin Shen, Feng Yu, Jun-chang Guan

**Affiliations:** 1 Department of Microbiology and Anhui Key Laboratory of Infection and Immunity, Bengbu Medical College, 2600 Dong Hai Avenue, Bengbu, Anhui 233030 People’s Republic of China; 2 Editorial Board of Journal of Bengbu Medical College, Bengbu Medical College, Bengbu, Anhui 233030 People’s Republic of China; 3 Department of Life Sciences, Bengbu Medical College, Bengbu, Anhui 233030 People’s Republic of China; 4 Scientific Research Center, Bengbu Medical College, Bengbu, Anhui 233030 People’s Republic of China; 5 Huzhou University Schools of Medicine and Nursing Science, Huzhou, Zhejiang 313000 People’s Republic of China

**Keywords:** Staphylococcal enterotoxin B, Splenic lymphocyte, Pregnancy, Offspring, Rat

## Abstract

**Background:**

Our previous study suggested that SEB exposure in pregnant rats could lead to the change of T cells subpopulation in both peripheral blood and thymus of the offspring rats. However, rarely is known about the influence of SEB exposure in pregnant rats on T cell subpopulation in the spleens of offspring rats.

**Results:**

SEB was intravenously administered to the pregnant rats at gestational day 16 in this study. The percentages, in vivo and in vitro responses of CD4 and CD8 T cells were investigated with flow cytometry. The prenatal SEB exposure obviously increased splenic CD4 T cell percentages of both neonates and adult offspring rats, and obviously reduced splenic CD8 T cell percentages of both the fifth day neonates and adult offspring rats. After spleens in the adult offspring rats were re-stimulated with SEB in vivo or in vitro, in vivo SEB stimulation could lead to the marked decrease of splenic CD4 T cell percentage and the marked increase of splenic CD8 T cell percentage. While in vitro SEB stimulation to the cultured splenocytes markedly decreased the proliferation of the splenic lymphocytes and the CD4 T cell percentage, and had no influence on CD8 T cell percentage.

**Conclusion:**

The prenatal SEB exposure could alter the percentages of CD4/CD8 T cell subpopulation and the response of CD4 and CD8 T cells to the in vivo and in vitro secondary SEB stimulation in the splenocytes of adult offspring rats.

## Background

In five types (A to E) of staphylococcal enterotoxin [1], staphylococcal enterotoxin B (SEB) as an important superantigen (SAg) has been widely studied. SEB can cross-link major histocompatibility complex class II molecules on the antigen-presenting cell with the β chain of the T cell receptor and activate vigorous fractions of the T cell population at high frequency [2, 3], which has no need of classical processing and presentation of antigen [4]. The immune response of T cells to SEB displays a biphasic change [5, 6] which consists of an early activation presented as T cell proliferation and a second anergy due to apoptosis of the appropriate T cells. Ultimately, the hyper-response and immunosuppression of T cells following SEB exposure may lead to illness and disease in mammals [7, 8]. A variety of literatures [9–12] have demonstrated the influence of SEB exposure on T cells during adulthood or neonatal period in animal experiments. Our previous study suggested that SEB exposure in pregnant rats could influence the T cells subpopulation in both peripheral blood and thymus of the offspring rats [13–15], but rarely is known about how SEB exposure in pregnant rats to influence the splenic T cell subpopulation of offspring rats. Therefore, SEB was intravenously injected to the maternal rats at gestational day (GD) 16 in this study. The percentages, in vivo and in vitro responses of CD4/CD8 T cells to SEB were investigated with flow cytometry in the spleens of offspring rats born to maternal rats exposed SEB during pregnancy.

## Methods

### Animals

Three-month-old Sprague–Dawley rats used in this study were fed with rodent chow and filtered tap water ad libitum and housed under controlled conditions at 23 °C ±2 °C and a constant 12 h light/12 h dark cycle. Each female rat was placed in contact with an adult male rat for mating. After 15 h, a plug was evaluated in female rats. The day of the plug observed initially in the vagina was considered day 1 of gestation (GD). Then, the female rats were kept in separate cages and randomly separated into the phosphate buffer saline (PBS) group and the SEB group. Twelve pregnant rats (four of them used in our previous papers [13, 15]) were used for present study in each group. In the SEB group, the pregnant rats were injected i.v. once with 0.3 ml 50 μg/ml SEB (Sigma-Aldrich, St Louis, MO) in 0.2 M PBS. The pregnant rats in PBS group were injected with the same volume of PBS. Then, the pregnant rats were reared as above and allowed to give birth naturally. Some neonatal offspring rats between days 0 and 5 after delivery were used to analyze T cell subpopulation in the spleens, the others were fed to adult offspring rats (about 3 to 5 months) for the analysis of T cell subpopulation and lymphocyte proliferation in the spleens. All surgery was performed under sodium pentobarbital anesthesia, and euthanasia was accomplished with CO_2_.

### In vivo response of adult offspring rats to SEB re-stimulation

When neonates were fed to adult offspring rats in the PBS and SEB groups, the adult offspring rats were administrated with either SEB or PBS in the same way as pregnant rats and continued to rear for 5 days. Then, the rats were anaesthetized and spleens were harvested to prepare for the splenocyte suspensions.

### Preparation of splenocyte suspensions

The spleens of the neonatal and adult offspring rats were minced and pressed through a 100-μm fine wire mesh screen. Cells mixture with PBS were collected in 5 ml centrifugal tube and centrifuged at 400 g for 10 min at 4 °C. After removing the supernatant, cell pellet was acquired and added with lysis buffer (Beyotime Biotechnology, China) to break red blood cells, according to the manufacturer’s instructions. Then, the cells were washed three times with balanced PBS solution containing 2% fetal bovine serum (FBS) (HyClone, UT) by centrifugation at 400 g for 10 min at 4 °C. Finally, splenocytes were mixed in staining buffer (PBS containing 2% FBS and 0.02% NaN3) and counted 1 × 10^6^/ml cells for detecting T cell subpopulation.

### Splenic lymphocyte culture

The spleens of the adult offspring rats in two groups were aseptically removed and splenic lymphocytes were isolated by standard Ficoll–Paque density gradient from each animal. The cells (1 × 10^5^/ml) were co-cultured with either 100 ng/ml SEB or 5 μg/ml concanavalin (Con) A (Sigma-Aldrich, St Louis, MO) for 3 days in 96-well and 24-well culture plates in 200 μl RPMI medium (Gibco BRL, USA) containing 10% FBS, L-glutamine, penicillin and streptomycin in a humidified incubator in 5% CO2 at 37 °C. For analysis of the T cells subpopulation, the lymphocytes in 24-well culture plates were harvested at the end of culture days 1, 2, & 3 and stained for the analysis of T cell subpopulation with flow cytometry as shown below. For the experiment of the lymphocyte proliferation, 1 μCi ^3^H-thymidine was added to the lymphocytes in 96-well culture plates 5 h before the end of culture days 1, 2, & 3. Thymidine incorporation by cells was determined using a cell harvester and 1450 MICROBETA liquid scintillation counter (PerkinElmer®, Waltham, MA, USA). Proliferation was measured as radioactivity incorporation [presented as counts per minute (CPM)].

### Flow cytometry cell analysis

Splenocyte suspensions were incubated and stained with fluorescently labeled antibodies of anti–CD3-FITC, anti–CD4-APC, anti–CD8a-PE (eBioscience, USA) in the dark at room temperature for 30 min. For determination of T cell subpopulation, flow cytometry was performed on a FACS calibur (Becton Dickinson, Heidelberg, Germany). Images of stained cells were analyzed with CellQuest analysis software (BD Biosciences, Franklin Lakes, NJ, USA).

### Statistical analysis

Statistical analysis was performed with SPSS software. To evaluate the difference of splenic T cells in the adult offspring rats, independent *T*-test was used. Turkey’s-b in one-way ANOVA was employed to evaluate the different significances of T cells in the neonatal offspring rats, as well as the proliferation and response of splenocytes re-stimulated with SEB in the adult offspring rats. Data are expressed as the mean ± SEM. Statistical significance was defined as *p* < 0.05.

## Results

### Influence of SEB exposed prenatally on splenic CD4/CD8 T cells of offspring rats

**Fig. 1:**
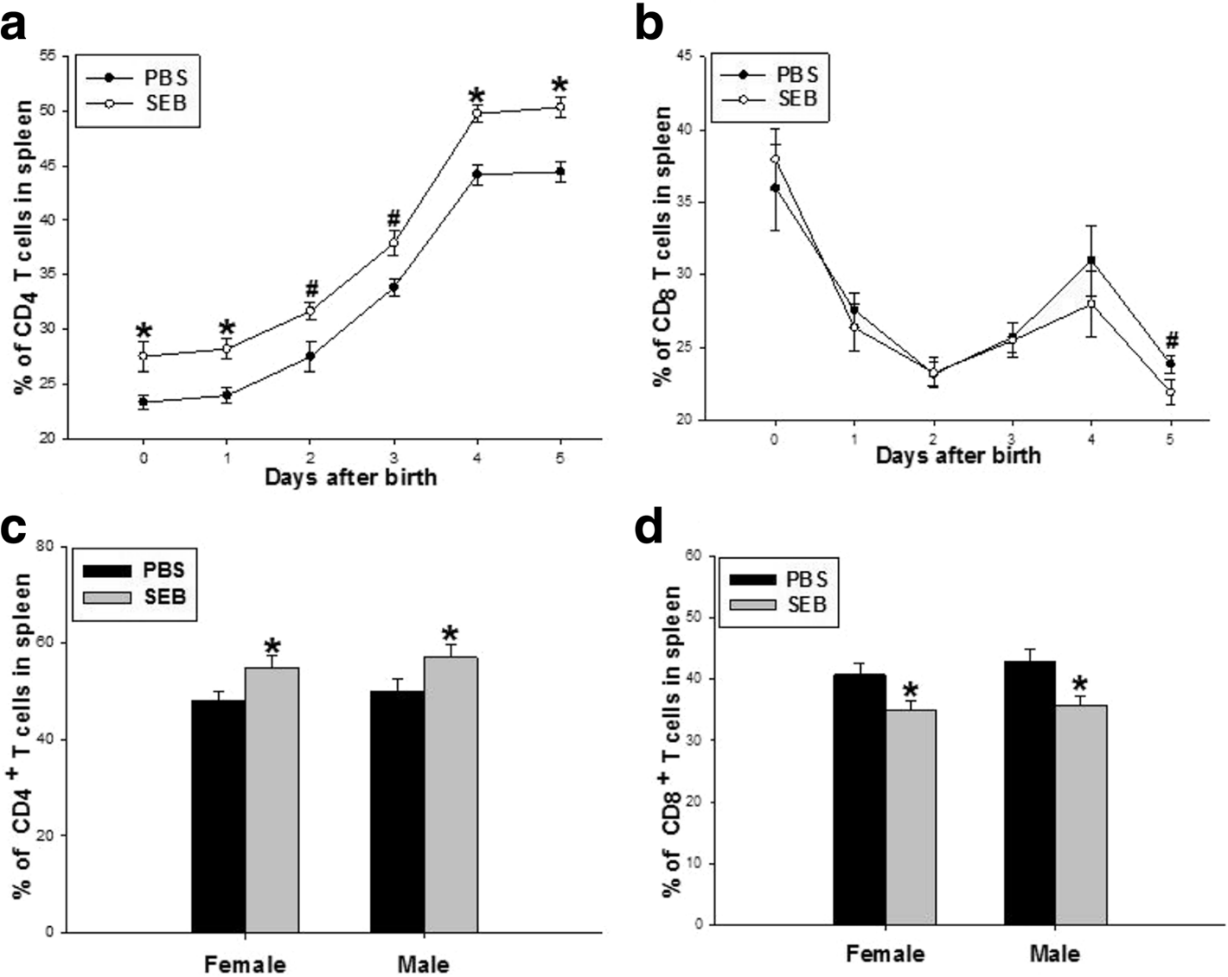
Effect of prenatal SEB exposure on CD4/CD8 T cells in the spleens of offspring rats. The spleens of the neonatal rats between days 0 and 5 after delivery and the adult offspring rats were harvested in the PBS and SEB groups. The percentages of both CD4 and CD8 T cells in the spleens of the neonatal rats (**a**, **b**) and the adult offspring rats (**c**, **d**) were analyzed by flow cytometry. Values were calculated with data from 12 independent experiments [In each group, 72 of neonatal offspring rats and 24 of adult offspring rats (half male and half female) were used.]. Each experiment of the neonatal rats included 1–2 neonatal rats of the same litter from same mother. Data represent mean ± SE. Compared with the PBS group at each time point: # *P* < 0.05; * *P* < 0.01

Compared with the PBS group, CD4 T cell percentage was obviously increased in the spleens of neonatal rats between days 0 and 5 after delivery (Fig. 1a), while CD8 T cell percentage was significantly decreased in the fifth-day neonates in the SEB group, but not different between the PBS and SEB groups between days 0 and 4 after delivery (Fig. 1b). In the adult offspring rats, it was revealed that the prenatal SEB exposure caused the markedly increased percentage of splenic CD4 T cells (Fig. 1c) and the markedly reduced percentage of splenic CD8 T cells (Fig. 1d).

### In vivo response of CD4/CD8 T cells to SEB re-stimulation

**Fig. 2:**
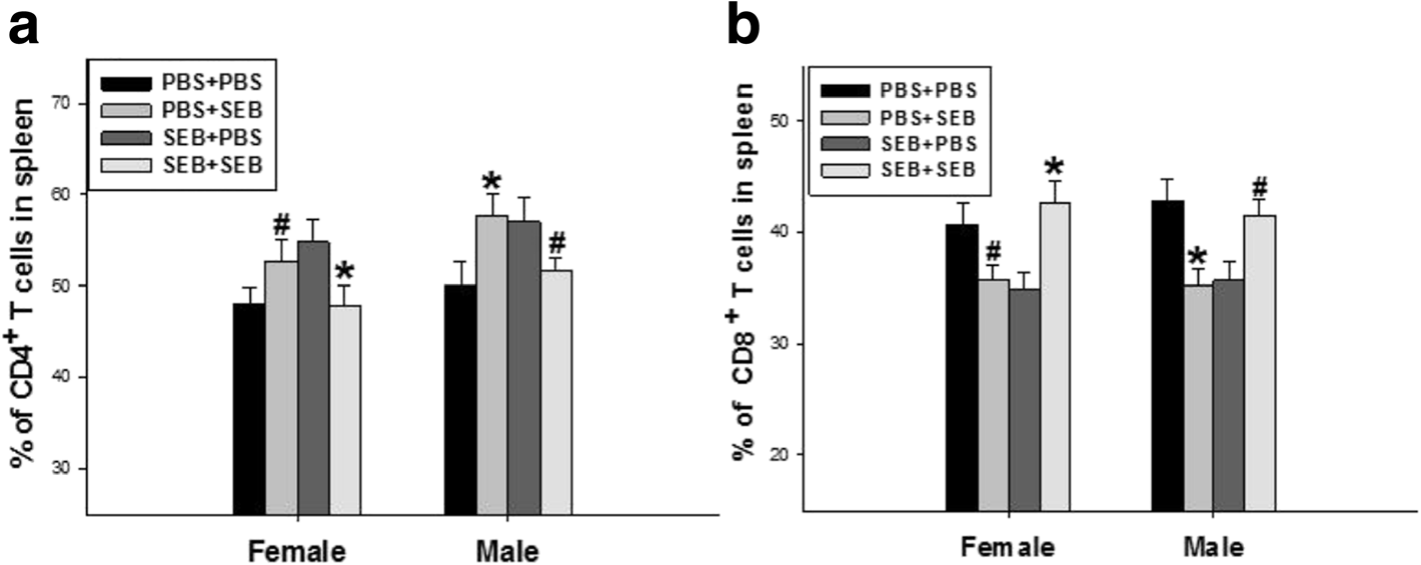
Effect of the secondary SEB administration on CD4/CD8 T cells in the spleens of adult offspring rats exposed prenatally to SEB. The adult offspring rats in the PBS and SEB groups were injected i.v. with either SEB (named as PBS + SEB, SEB + SEB) or PBS (named as PBS + PBS, SEB + PBS), separately. Five days after administration, the splenocytes of adult female and male offspring rats were harvested. The percentages of both CD4 (**a**) and CD8 (**b**) T cells were analyzed by flow cytometry. Values were calculated with data from 10 independent experiments [Twenty adult offspring rats (half male and half female) was used in each group.]. Data represent mean ± SE. Compared with PBS + PBS: # *P* < 0.05; Compared with SEB + PBS: * *P* < 0.05

Five days after in vivo PBS or SEB administration to the adult offspring rats, the percentage of T cell subpopulation in the spleens were detected with flow cytometry. In the PBS group, it indicated that SEB administration led to the markedly higher percentage of CD4 T cells (Fig. 2a) and the markedly lower percentage of CD8 T cells (Fig. 2b) than those of PBS administration in the adult offspring rats. While the response trends of CD4 (Fig. 2a) and CD8 (Fig. 2b) T cells to secondary SEB administration in the SEB group were completely contrary to those in the PBS group.

### In vitro response of the splenic lymphocytes of adult offspring rats to SEB

**Fig. 3:**
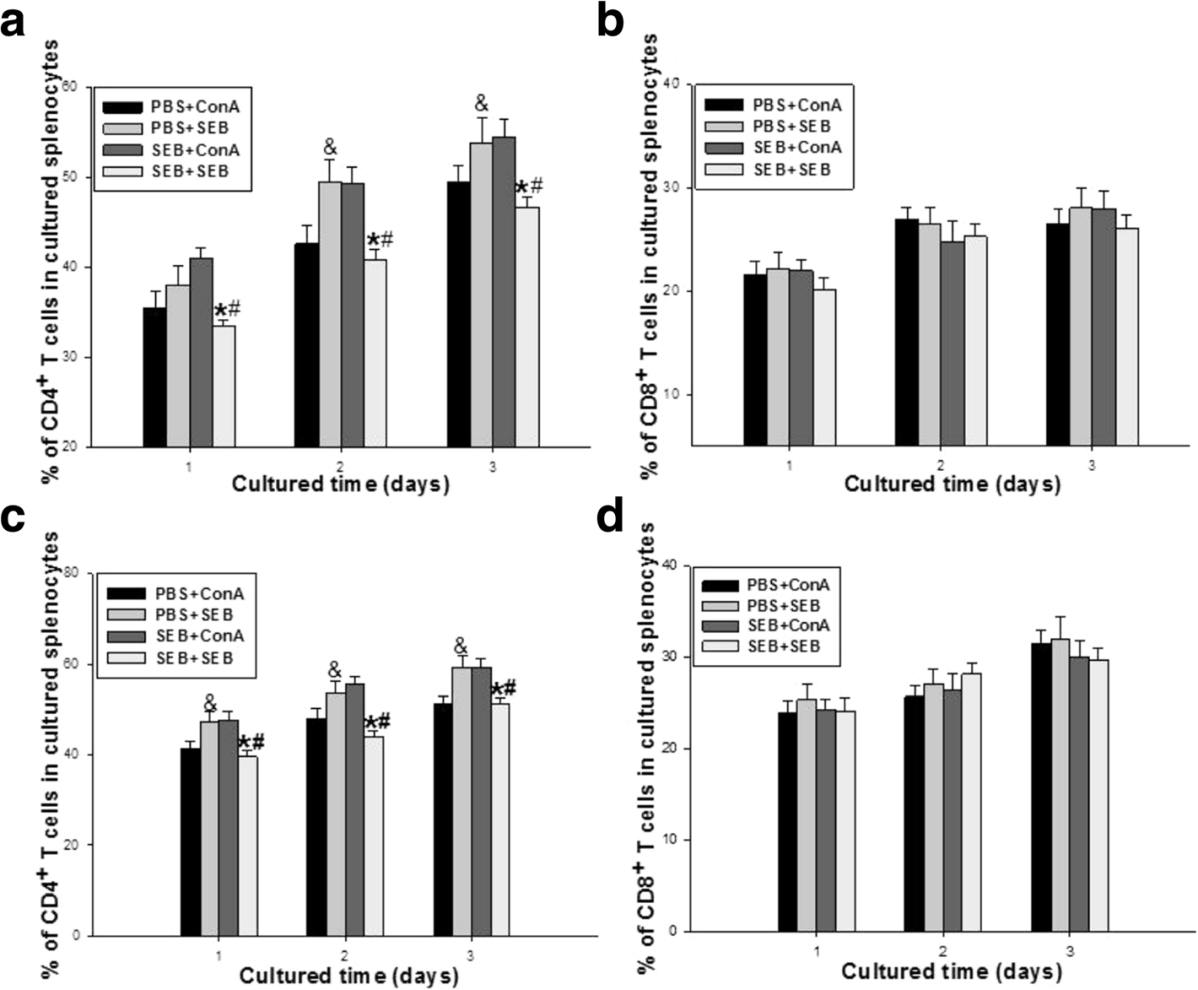
In vitro response of the splenic lymphocytes of adult offspring rats to SEB. After the splenic lymphocytes of adult offspring rats were acquired in the PBS and SEB groups and in vitro co-cultured in RPMI medium with either SEB (named as PBS + SEB, SEB + SEB) or ConA (named as PBS + ConA, SEB + ConA) for 3 days, the percentages of CD4 and CD8 T cells in the splenocytes of adult female (**a**, **b**) and male (**c**, **d**) offspring rats were analyzed by flow cytometry. Values were calculated with data from 10 independent experiments [Twenty adult offspring rats (half male and half female) was used in each group.]. Data represent mean ± SE. Compared with PBS + ConA: & *P* < 0.05; Compared with SEB + ConA: * *P* < 0.05; Compared with PBS + SEB: # *P* < 0.05

After the splenic lymphocytes of adult offspring rats were in vitro co-cultured with either SEB or ConA for 3 days, the percentages of CD4 and CD8 T cells were examined. In the PBS group, the CD4 T cell percentage with SEB stimulation was markedly higher than those with ConA stimulation in each cultured day (Fig. 3a, b). While in the SEB group, in vitro SEB stimulation markedly reduced the CD4 T cell percentage compared with that with ConA stimulation. However, neither SEB nor ConA stimulation in each cultured day altered the percentage of CD8 T cells in the PBS and SEB groups (Fig. 3b, d).

### Proliferation of the splenic lymphocytes

**Fig. 4:**
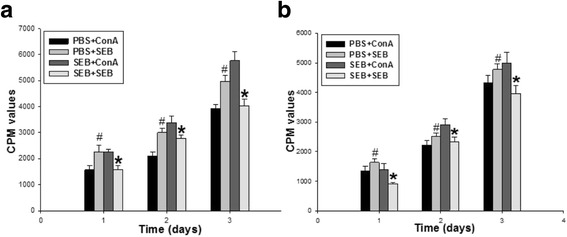
Effect of SEB on the lymphocyte proliferation of adult offspring rats. Three days after the splenocytes of adult offspring rats in the PBS and SEB groups were in vitro co-cultured in RPMI medium with either SEB (named as PBS + SEB, SEB + SEB) or ConA (named as PBS + ConA, SEB + ConA), the proliferation was measured by ^3^H-thymidine incorporation in the splenic lymphocytes of adult female (**a**) and male (**b**) offspring rats. Data are expressed as mean ± SE and the measure unit of [^3^H] thymidine incorporation is counts per min (CPM). The results are representative of 10 independent cultures with each condition in triplicate [Twenty adult offspring rats (half male and half female) was used in each group]. Compared with PBS + ConA: # *P* < 0.05; Compared with SEB + ConA: * *P* < 0.05

After in vitro co-cultured with either SEB or ConA for 3 days, proliferation of the splenic lymphocytes of adult offspring rats was measured by ^3^H-thymidine incorporation. With the increase of stimulation time, the proliferation of lymphocytes stimulated by SEB and ConA was significantly increased in the lymphocytes of adult female (Fig. 4a) and male (Fig. 4b) offspring rats in the PBS and SEB groups. While in the PBS group, SEB stimulation significantly increased the lymphocyte proliferation compared with that stimulated by ConA in each cultured day, but in the SEB group, the proliferation of lymphocytes stimulated by SEB was significantly lower than that by ConA in each cultured day.

## Discussion

In this study, the data indicated that SEB exposure in pregnant rats markedly altered the percentages of both CD4 and CD8 T cells, as well as the response characteristics of CD4/CD8 T cells to secondary SEB administration, not only in vivo but also in vitro, in the splenocytes of adult offspring rats. As far as we know, the present study firstly investigates the effect of SEB exposure in pregnant rats on the changes and responses in splenic T cell subpopulation of offspring rats.

SEB exposure to naive mice could selectively lead to specific anergy of Vβ8 T cells [16–18] of not only the peripheral but also central immune compartments [5, 19]. Although our previous study [15] suggested that SEB exposure in pregnant rats could affect thymic T cell subpopulation in the central compartment of the offspring rats, rarely is known about the influence of SEB exposure on T cell subpopulation in the spleens. The present study showed that SEB exposure in pregnant rats could cause the increased percentage of splenic CD4 T cells between days 0 and 5 after delivery and the decreased percentage of splenic CD8 T cells in the fifth day in the neonatal offspring rats. While in the spleens of adult offspring rats, SEB exposure in pregnant rats was able to lead to the increased CD4 T cells and the decreased CD8 T cells, in accordance with other data from direct SEB stimulation in adult mice [5, 20]. These results suggest that SEB exposure in pregnant rats was able to cause the decreased percentage of CD8 T cells accompanied by a relative increase in the percentage of CD4 T cells and could imprint the changes of the CD4 and CD8 T cell percentage from neonatal to adult offspring rats. The trend of these changes was similar to those of both CD4 and CD8 T cells in the thymus [15] and peripheral blood [13] of offspring rats in our previous study, suggesting the consistency of changes between central and peripheral compartments about the effect of prenatal exposure of SEB on the CD4 and CD8 T cells. Many studies [21, 22] have suggested that the imprinting effects are caused by the physicochemical and biological factors exposed during pregnancy, and are the developmental origins of health and disease. A lot of studies indicate that SEB exposure is involved in the pathogenicity of some immunological diseases [9, 23]. Whether the imprinted alteration of T cells caused by prenatal SEB exposure is associated with these diseases remains further study in the future. In addition, whether male or female adult offspring rats, there had no difference of splenic T cell percentage between PBS and SEB groups. These data suggest the effect of SEB exposure in pregnant rats on T cell subpopulation did not display gender difference.

Since prenatal SEB exposure could lead to the changes of splenic T cells in the offspring rats, another question raised immediately was whether prenatal exposure of SEB could influence T cell function in the spleens of the offspring rats. To address this question, the responses of splenocytes to the in vivo and in vitro secondary SEB stimulation were further investigated in the adult offspring rats. Five days after the in vivo secondary SEB administration to the adult offspring rats exposed SEB during pregnancy, interesting results were found that the SEB re-stimulation significantly reduced CD4 T cells with an increased CD8 T cell percentage in the spleen of the adult offspring rats, which was contrary to the results from the primed SEB administration during pregnancy in the adult offspring rats. It may be due to the possibility that the primed SEB administration during pregnancy abrogated the response of CD4 T cells (anergy) despite these cells existed in significant percentage [17, 24]. Furthermore, the SEB administration to the in vitro cultured splenocytes in the adult offspring rats was also able to induce the significantly decreased CD4 T cell percentage compared with that of the ConA administration, but had no influence on the CD8 T cell percentage. These data suggest that SEB exposure in pregnant rats could lead to the hypo-responsiveness or anergy of CD4 T cells in the splenocytes of adult offspring rats, which is consistent with other results from direct SEB administration in the cultured splenocytes of the adult mice [17, 25, 26]. Taken together, these data suggest that SEB exposure in pregnant rats could alter the secondary response of CD4 /CD8 T cells to both in vivo and in vitro SEB re-stimulation in the splenocytes of adult offspring rats.

## Conclusions

SEB exposure in pregnant rats could induce the decrease in the percentage of CD8 T cells accompanied by a relative increase in the percentage of CD4 T cells and could imprint the changes of the CD4 and CD8 T cell percentages from the neonatal to adult offspring rats. Furthermore, SEB exposure in pregnant rats could alter the secondary response of CD4 and CD8 T cells to both in vivo and in vitro SEB re-stimulation in the splenocytes of adult offspring rats.

## Abbreviations

Con A: Concanavalin A
CPM: Counts per minute
FBS: Fetal bovine serum
GD: Gestational day
PBS: Phosphate buffer saline
SAg: Superantigen
SEB: Staphylococcal enterotoxin B
